# Antioxidant and Anti-Inflammatory Actions of Polyphenols from Red and White Grape Pomace in Ischemic Heart Diseases

**DOI:** 10.3390/biomedicines10102337

**Published:** 2022-09-20

**Authors:** Ioana Corina Bocsan, Dan Claudiu Măgureanu, Raluca Maria Pop, Antonia Mihaela Levai, Ștefan Octavian Macovei, Ioana Maria Pătrașca, Veronica Sanda Chedea, Anca Dana Buzoianu

**Affiliations:** 1 Faculty of Medicine, Department of Pharmacology, Toxicology and Clinical Pharmacology, “Iuliu Hatieganu” University of Medicine and Pharmacy, No. 23, Marinescu Street, 400012 Cluj Napoca, Romania; 2 Faculty of Medicine, “Iuliu Hatieganu” University of Medicine and Pharmacy, 400012 Cluj Napoca, Romania; 3 Faculty of Medicine, Department Mother and Child, “Iuliu Hatieganu” University of Medicine and Pharmacy, No. 3-5, Clinicilor Street, 400012 Cluj Napoca, Romania; 4 Research Station for Viticulture and Enology Blaj (SCDVV Blaj), 515400 Blaj, Romania

**Keywords:** antioxidant, anti-inflammatory, grape pomace, polyphenols, ischemic heart diseases

## Abstract

Grape pomace (GP) represents a very reliable source of polyphenols because it could be found globally as a remnant of the wine industry. During the winemaking process, two types of GP are generated: red GP and white GP, according to the produced wine, red or white. Grape pomace represents a viable source of polyphenols, mainly flavanols, procyanidins anthocyanins, and resveratrol which possess antioxidant and anti-inflammatory activities. Multiple differences were observed between red and white GP in terms of their antioxidant and anti-inflammatory activity in both in vitro and in vivo studies. Although most studies are focused on the antioxidant and anti-inflammatory effect of red grape pomace, there are still many variables that need to be taken into consideration, as well as extensive study of the white GP. It was observed that in both in vitro and in vivo studies, the GP polyphenols have a direct antioxidant activity by acting as a free radical scavenger or donating a hydrogen atom. It also possesses an indirect antioxidant and anti-inflammatory activity by reducing mitochondrial reactive oxygen species (ROS) generation, malondialdehyde (MDA), tumor necrosis factor-alpha (TNF-α), interleukin-1-beta (IL-1β), interleukin-6 (IL-6), nuclear factor kappa-light-chain-enhancer of activated B cells (NF- κβ), and inhibitor of nuclear factor kappa-B kinase subunit beta (Iκκβ) levels or nitrate oxide-4 (NOX4) expression and by increasing the levels of antioxidants enzymes like superoxide dismutase (SOD), catalase (CAT) glutathione reductase (GRx) and glutathione peroxidase(GPx). Besides these activities, many beneficial effects in ischemic heart diseases were also observed, such as the maintenance of the ventricular function as close as possible to normal, and the prevention of infarcted area extension. In this context, this review intends to present the actual knowledge of grape pomace’s potential antioxidant and anti-inflammatory activity in ischemic heart disease, knowledge gathered from existing in vitro and in vivo studies focused on this.

## 1. Introduction

Ischemic heart diseases, also known as coronary heart diseases (CAD), alongside stroke and other cardiovascular diseases, are the causes of approximately 17.9 million deaths annually, which represents 32% of the total deaths in the world [1]. Out of these, more than 75% are registered in low and middle-income countries. Furthermore, in accordance with World Health Organization (WHO), ischemic heart disease is the leading cause of global death, with 16% of worldwide deaths, followed by stroke, which is responsible for 11%, respectively. Ischemic heart disease is characterized by narrowing or blockage of one or more coronary arteries, most frequently due to atherosclerosis, which is the main factor that reduced cardiac blood flow. It is clinically manifested by pectoral angina and heart attack [2]. The main incriminated risk factors that promote CAD are tobacco, an unhealthy diet with low fruit and vegetable intake, lack of physical activity, metabolic syndrome, and excessive use of alcohol [1,3]. Besides these, other pathologies like obesity, diabetes mellitus, nephrotic syndrome, and hypothyroidism could associate with dyslipidemia, which is characterized by elevated levels of LDL and total cholesterol and a reduced HDL level. Moreover, it was observed that people with different lifestyles, like workers who have permanent night shifts, are more likely to develop dyslipidemia [4].

All of these risks lead to atherosclerosis. In this term, atherosclerosis is defined as a multifactorial inflammatory disease of the innermost layer of an artery called intima, a build-up of cholesterol plaque, and a loss of the arterial wall elasticity [5]. Therefore, a primary target in the treatment of CAD is represented by the prevention of atherosclerosis development. In this regard, the management of CAD includes lifestyle changes like dietary modification, smoking cessation, and weight reduction alongside classical medication (nitrates, beta-blockers, calcium channel blockers, and angiotensin-converting enzyme inhibitors). Additional comorbidities like diabetes, hypertension, and dyslipidemia are controlled via oral antidiabetics or insulin, antihypertensive drugs, and statins, respectively.

**Figure 1:**
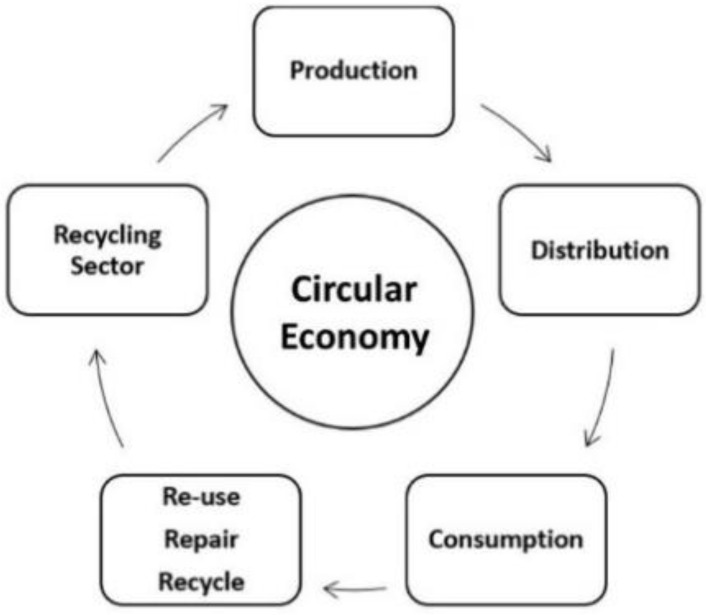
Circular Economy.

Even if there are many efficient ways of reducing the incidence of associated risk factors, among which the pharmacological and surgical ones with proven results, CAD still represents the main cause of death worldwide. That is why this pathology represents a great interest for many researchers and their efforts are needed in identifying new ways to prevent and treat CAD. In this regard, plants have been always an inexhaustible source of discovering new compounds with potent pharmacological activities. Shifting from traditional plant utilization, a great alternative is represented by plant waste valorization. This new direction came along with the introduction of the circular economy, an economic system that proposes a reduced use of raw materials and increased reuse and recycling of different components and products already existing [6] (Figure 1).

A perfect example of the circular economy’s application is represented by the usage of grape pomace (GP). Therefore, it is estimated that annually are used more than 79 million tons of grapes, from which approximately 30% is represented by grape pomace [7,8]. Besides its use as fertilizer or animal feed, another field in which it can be used is the pharmaceutical one, due to the rich amount of bioactive compounds, especially phenolic ones [8]. Thereby, GP is reported to contain high quantities of resveratrol and polyphenols like flavanols: myricetin, quercetin, kaempferol; flavan-3-ols: catechin, epicatechin; cinnamic acids: p-coumaric and benzoic acids: syringic, gallic, and protocatechuic, 4-hydroxybenzoic [9]. It is known that polyphenols, the major compounds in GP waste have well-known antioxidant and anti-inflammatory effects [10]. Previous studies have reported their action on reducing LDL oxidation, inflammation, and platelet activation, all with positive effects in reducing the progression of atherosclerosis [11].

In the present review, we aimed to evaluate the influence of grape pomace through its polyphenols on ischemic heart disease due to its antioxidant and anti-inflammatory activities.

## 2. Red and White Grape Pomace—Bioactive Compounds

**Figure 2:**
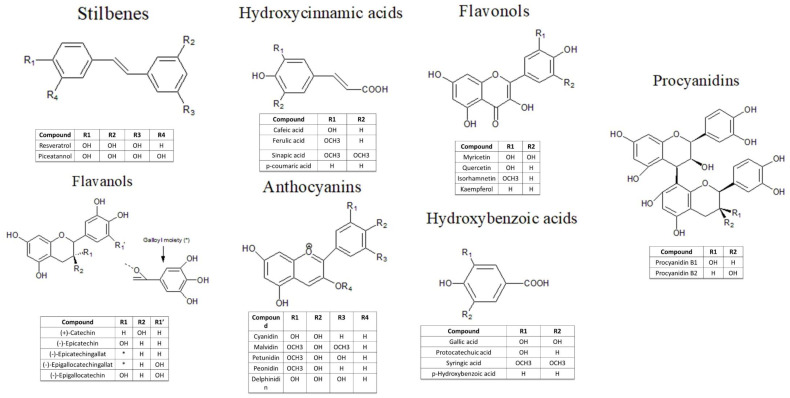
The chemical composition of principal polyphenols from grape pomace.

The utilization of grapes has a long history, which dates back to antiquity and spreads to the modern world, especially through their use in the wine industry. That is why there is a variety of literature studies that analyze and characterize grapes, grape derivatives especially wine, and GP composition and content [9,12] (Figure 2). It was observed that red grape pomace (RGP) and white grape pomace (WGP) have different phenolic compound fingerprints and different total phenolic content according to the grape cultivar and terroir. This means that all of the pedological, topographical, and geological aspects of a specific physical environment will alter the physical features of the grapes such as tastes, aromas, textures, and appearances [8]. RGP was found to be rich in stilbenes (resveratrol), phenolic acids (gallic acid, protocatechuic acid), flavanols (epigallocatechin), flavanols (myricetin-3-O-rhamnoside) and anthocyanins (delphinidil-3-O-glucoside, cyanidin-3-O-glucoside, petunidin-3-O-glucoside, peonidin-3-O-glucoside, malvidin-3-O-glucoside) [13]. WGP was reported to have high content of phenolic acids (p-hydroxyphenylacetic acid, vanillic acid, homovanillic acid, homoprotocatechuic acid, gentisic acid, syringic acid, 4-O-methylgallic acid, 3-O-methylgallic acid, dihydro-3-coumaric acid, hydroferulic acid, hydrocaffeic acid, isoferulic acid) and flavanols (catechin, epicatechin, procyanidin B1) [13], flavonoid glycoside (hyperoside, isoquercitrin, rutin, quercitrine), flavonoid aglycons (quercetin, luteolin), and protocatechuic acid [14]. It was also reported that WGP has a high quantity of gallic acid, procyanidin B3-4, epicatechin, and procyanidin gallates [15]. In both RGP and WGP was identified a similar amount of caffeic acid, coumaric acid, catechin, and its isomer epicatechin [14], and similar amounts of total tannins [16]. Overall, numerous studies concluded that RGP contains a higher amount of polyphenols than WGP [17]. However, some studies revealed types of WGP that possessed a greater content of polyphenols than RGP [13].

## 3. Potentially Toxic Effects of Polyphenols from Red and White Grape Pomace

Generally, literature studies focused on GP or GP by-products are emphasizing its health benefits rather than toxic or adverse reactions. Administration of GP may not be an issue in healthy people, but it must be considered in people with certain diseases who are receiving medical treatment [18]. Regarding the toxicity of polyphenols from grape pomace, we could find only one study. Thus, Neag et al., (2019) observed the paradoxical effect of GP polyphenol extract on an animal model of acute kidney injury induced by cisplatin. They reported that when GP, was given alongside cisplatin, for its antioxidant properties, it did not decrease the cisplatin-induced nephrotoxicity, on the contrary, it increased it [19]. The lack of studies reporting the potentially toxic effects of polyphenols from grape pomace could be based on the fact that even if GP presents pro-oxidant activity at a higher dose than the one that presents the antioxidant effect [20,21], that effect is too low to cause changes at the level of an organ or the entire organism, changes that could be highlighted through routine analyses. Thus, this issue should be addressed in terms of precautions rather than acute or chronic toxicity [22].

## 4. Red and White Grape Pomace—Variability of Total Polyphenols Content and Antioxidant Capacity

It is well-known that GP possesses great antioxidant activity, but it is necessary to find out what are the differences between RGP and WGP to give them an appropriate valorization. Literature studies showed that GP has a strong antioxidant activity, because of the contained phenolic compounds. The antioxidant activity is strongly related to phenolic chemical structures. Thus, the number of existing hydroxyl groups gives them the ability to act as free radical scavengers [23] or to donate an atom of hydrogen [24,25]. Accordingly, several methods for antioxidant activity evaluation have been created over time. The main methods used for GP characterization reported so far were total polyphenol content (TPC), 2,2-Diphenyl-1-picrylhydrazyl (DPPH), 2,2′–azinobis-(3-ethyl-benzothiazoline-6-sulphonic acid) (ABTS), and ferric reducing antioxidant power assay (FRAP) for antioxidant activity (Table 1). 

Total phenolic content represents the reference assay for measuring the polyphenols in plants or other biological samples, by using the Folin–Ciocalteu assay [24,26]. This method involves a reaction between the polyphenols and a redox reagent. Accordingly, the phenolic content is determined using the spectrophotometric measurement of this reaction [27]. Further, the methods used to determine polyphenols’ antioxidant activity content are ABTS, DPPH, and FRAP. These assay methods analyze the antioxidant activity via the donation of a hydrogen atom (ABTS and DPPH) or via electron transfer (FRAP) [28,29].

Due to the variety of antioxidant activity methods that are used, it is very difficult to compare data from the literature. This situation leads to the development of a relevant correlation method, which could allow their comparison. In this case, Xu et al., (2016), in their study regarding the phenolic compounds extracted from four GP varieties, identified antioxidants and compounds with antibacterial properties and also developed a correlation method between TPC, DPPH, and ABTS [30]. They observed that between TPC and ABTS there is a significant positive correlation, but none between these and DPPH. A probable cause for this could be the fact that there are differences between the phenolic compounds involved in each method. Thus, it was reported that flavonoids and tannins contributed to the determination of antioxidant activity via ABTS, while in the case of DPPH anthocyanins, they had a major contribution. In comparison to this study, Marchante et al., (2018) observed that in the measurement of antioxidant activity using the DPPH method, a higher contribution was brought by (−)-epigallocatechin, while in the case of the ABTS method by flavan-3-ol monomers. Furthermore, they did not observe any differences between the contribution to the determination of ABTS and DPPH methods for (+)-catechin, (+)-gallocatechin, (+)-epigallocatechin, (+)-catechin gallate, (−)-epicatechin gallate, procyanidin B1, galloylated dimers, flavan-3-ol dimers, flavan-3-ol total oligomers, total flavan-3-ols, and trans-resveratrol-glucoside. Moreover, they also observed that the compound with the highest antioxidant property was (+)-catechin gallate, followed by (−)-epicatechin gallate, (+)-gallocatechin, (+)-catechin, and (−)-epigallocatechin [31].

Xia et al., (2019), also addressed the necessity of comparing and correlating the values of the different methods used in polyphenols quantification. To eliminate the variations of these values, the authors also chose to determine the antioxidant activity by using all the above-described methods. Thus, Xia et al., (2019), evaluated the TPC and measured the antioxidant assay using ABTS, DPPH, and FRAP of skin and seeds from 31 different cultivars of grapes. Firstly, they observed that the grape seeds have more polyphenols and more antioxidant activity as assayed via DPPH, ABTS, and FRAP than grape skins. Secondly, they observed that the European species have higher antioxidant properties than the American, Asian, or hybrid ones [10].

Even though the majority of studies determined that RGP possesses a higher polyphenolic content and antioxidant activity (Costa et al., 2018, Sagdic et al., 2011, Xu et al., 2016), there is no sufficient evidence yet to affirm that RGP is superior to WGP. Winkler et al., (2015) observed that even though the RGP cultivated in Rhineland-Palatinate, Germany had a higher TPC than WGP, the differences were not significant [32]. Further, Cerda-Carrasco et al., (2015) who investigated GP obtained from *Vitis vinifera* sp. cultivated in Maipo Valley, Chile, observed that two types of white grapes, Sauvignon Blanc and Chardonnay, had higher phenolic content and antioxidant capacity than the two red types, Cabernet Sauvignon and Carménère [15].

Knowing that the differences between RGP and WGP are influenced by the wine-making technologies [15] and also by the terroir [8,32], we can highlight the importance of a correlation method that could help in the appropriate GP waste valorization. To emphasize these differences, the next table presents the variation of TPC and antioxidant capacity identified in different GP varieties.

We can conclude that both GP varieties represent great sources for further valorization, their prior analysis being a key step in directing toward the appropriate use, because of their large variation in terms of phenolic content and antioxidant activity.

**Table 1:** Comparison of total polyphenols content (TPC) and antioxidant capacity of red and white grape pomace polyphenols extracts.

Grape Pomace (GP)		TPC (mg GAE */g GP)	Antioxidant Capacity			References
			DPPH (μmol TE **/g GP)	ABTS (μmol TE/g GP)	FRAP (μmol F_e_SO_4_ * 7H_2_O/g GP)	
*Vitis vinifera* sp. Cultivated in Maipo Valley, Chile						[15]
White	Sauvignon Blanc	19	120	-	-	
	Chardonnay	17	90	-	-	
Red	Cabernet Sauvignon	14	60	-	-	
	Carménère	13	70	-	-	
*Vitis vinifera* sp. cultivated in Virginia, USA						[30]
White	Vidal Blanc (hybrid variety)	55.5	7.71	334	-	
	Viognier (*Vitis vinifera* sp.)	99.1	3.54	951	-	
Red	Cabernet Franc (*V. vinifera* sp.)	153.8	11.2	1013	-	
	Chambourcin (hybrid variety)	92.0	28.2	378	-	
*Vitis vinifera* sp. cultivated in Rhineland-Palatinate, Germany						[32]
White	4 varieties of Pinot Blanc and 6 of Riesling	48	-	-	-	
Red	5 varieties of Dornfelder, 5 of Pinot noir and 2 of Portugais bleu	58	-	-	-	
*Vitis vinifera* sp. cultivated in Blacksburg, Crozet, Floyd VA, USA						[33]
White	Viognier	11.8	-	-	-	
	Vidal Blanc	12.5	-	-	-	
	Niagara	24.8	-	-	-	
	Petit Manseng	32.1	-	-	-	
Red	Petit Verdot	64.8	-	-	-	
	Merlot	35.8	-	-	-	
	Cabernet Franc	36.1	-	-	-	
	Chambourcin	10.4	-	-	-	
White	unknown varieties	90.51	-	-	1619	[34]
Red	unknown varieties	107.40	-	-	1886	
*Vitis vinifera* sp. cultivated in Cappadocia district of Nevsehir province (Emir), Tokat province (Narince), Sarkoy-Murefte district of Trakya region (Gamay), Ankara province (Kalecik Karasi), Elazig province (Okuzgozu), Turkey						[35]
White	Emir	75.5	-	-	-	
	Narince	138.1	-	-	-	
Red	Gamay	255.4	-	-	-	
	Kalecik Karasi	205.7	-	-	-	
	Okuzgozu	281.4	-	-	-	
*Vitis vinifera* sp. cultivated in Blackstone, VA, USA						[36]
White	Chardonnay	24.5	-	-	-	
Red	Cabernet Franc	30.4	-	-	-	
*Vitis vinifera* sp. cultivated in Cantine Cantele, Apulia Region, Southern Italy						[37]
White	Fiano	127.06	-	-	-	
Red	Negramaro	127.87	-	-	-	
*Vitis vinifera* sp. cultivated in Paros, Greece						[38]
White	Monemvassia	4.49	-	-	0.32	
Red	Mandilaria	5.1	-	-	0.31	
	Aidani mavro	0.25	-	-	0.21	

* GAE—gallic acid equivalent ** TE—Trolox equivalent.

## 5. Red and White Grape Pomace—In Vitro Antioxidant and Anti-Inflammatory Activities

The fact that many studies investigating GP have shown that it possesses intense antioxidant activity has been a key factor in drawing the attention of researchers to continue the findings and to focus on the beneficial antioxidant and anti-inflammatory activities within the in vitro studies as presented in Table 2. Most literature data reports the antioxidant activity of GP on cells exposed to different oxidative stress factors or/and the anti-inflammatory activity on cells subjected to different proinflammatory factors. 

### 5.1. Red Grape Pomace Antioxidant Activity

Within the in vitro models of oxidative stress induced in different cell lines, hydrogen peroxide (H_2_O_2_), tert-butyl hydroperoxide, and menadione are the most used chemicals. Physical factors, especially UV radiation, are also often reported. Accordingly, Posadino et al., (2018) studied the antioxidant activity of RGP from *Vitis vinifera* L. Cagnulari cv. from Santa Maria La Palma, Alghero, Italy on H_2_O_2_-induced oxidative damage in human umbilical vein endothelial cells. They observed that under oxidative stress conditions, the treatment with RGP increased the viability of the cells, mainly due to RGP’s capacity to reduce ROS levels [39]. Goutzourelas et al., (2014) investigated the antioxidant capacity of RGP from *Vitis vinifera* L. Batiki Tyrnavou cv. from Greece against oxidative stress. Within this study, the oxidative stress conditions were induced using tert-butyl hydroperoxide in muscle cells (C2C12) and endothelial cells (EA.hy926). The results obtained on muscle cells line indicated that RGP possesses high antioxidant activity demonstrated by TBARS (thiobarbituric reactive substances), ROS (reactive oxygen species), and protein carbonyl level reduction, and by increasing GSH (glutathione) levels. The results obtained from the endothelial cells line had similar results, except for the reduction of ROS levels [40]. The antioxidant effects of RGP from *Vitis vinifera* seeds were investigated by Decean et al., (2016) using UV radiation-induced oxidative stress in human keratinocytes cells (HaCaT cells). They observed that cells pre-treated with RGP had a significantly lower increase in ROS and protein levels that are specific to the apoptosis process. A lower increase in Bax-α pro-apoptotic protein and NF-kB p65 protein levels was also observed [41]. 

### 5.2. White Grape Pomace Antioxidant Activity

So far, in the literature, we have identified a few studies that investigated the antioxidant activity of WGP. Thereby, Bibi et al., (2017) studied the antioxidant activity of WGP from *Vitis vinifera* cv Chardonnay from Lowden, WA, USA on H_2_O_2_-induced oxidative damage in human colonic epithelial cells. They observed that WGP played a major role in reducing ROS levels [47]. Furthermore, WGP antioxidant activity was also analyzed in comparison with RGP by Domínguez-Perles et al., (2016). They studied two red types (Tinto Cão, Tinta Barroca) and two white types (Malvasia Fina, Moscatel Branco) from *Vitis vinifera* varieties from Quinta da Cavadinha, Pinhão, Portugal. They highlighted that, on H_2_O_2_-induced oxidative damage in human keratinocytes (HaCaT cells), all four types possessed antioxidant activity due to a significant decrease in ROS and LPO levels and a significant increase in GSH levels. Of all these four types, the highest decreases in LPO levels and the highest increases in GSH levels were recorded in the case of WGP from Malvasia Fina. On the other hand, the highest decreases in ROS levels were reported for RGP from Tinto Cão [48].

### 5.3. Red Grape Pomace Anti-Inflammatory Activity

Regarding experimental-induced inflammation in different cell lines in vitro, the most used chemicals are polycyclic aromatic hydrocarbons, TNF-α (tumor necrosis factor- α), and LPS (lipopolysaccharide). Thus, Pistol et al., (2019) investigated the anti-inflammatory activity of RGP from Vitis vinifera grown in Valea Călugărească, România. Within their study, RGP was used as a prebiotic in combination with Lactobacillus sp. used as a probiotic, on LPS-induced inflammation in human colorectal adenocarcinoma-derived intestinal epithelial cells (Caco-2) intestinal cells. This combination led to the down-regulation of the pro-inflammatory chemokines and cytokines genes’ expression, and an up-regulation of TIMP1 (matrix metalloproteinase inhibitors 1) and TIMP2 (matrix metalloproteinase inhibitors 1) expression [42]. Moreover, they observed the anti-inflammatory effect by down-regulation of many inflammations signaling pathways such as JNK1 (c-Jun N-terminal kinase), ERK1/2 (extracellular signal-regulated kinase ½), Akt/P70S6K/mTOR (Akt—protein kinase B, P70S6K—ribosomal protein S6 kinase, mTOR—mammalian target of rapamycin), MAPK (Mitogen-activated protein kinase), NF-κB (nuclear factor kappa-light-chain-enhancer of activated B cells), and Nrf2 (nuclear factor erythroid 2–related factor 2) [42]. In the study of Calabriso et al., (2022) the anti-inflammatory effects of RGP from *Vitis vinifera* L., cv Negramaro from Guagnano, Lecce, Italy was investigated on LPS and TNF-α-induced inflammation in Caco-2 cells and in human microvascular endothelial cells (HMEC-1 cells). Using the ELISA assay, they observed that the treatment with RGP induced a decrease in IL-6 (interleukin-6) and MCP-1 (monocyte chemoattractant protein-1) levels and down-regulation of MMP-9 (matrix metalloproteinases-9) and MMP-2 (matrix metalloproteinases-2) expression [45]. Moreover, they also highlighted the down-regulation of the NF- κB pro-inflammatory signaling pathways and the down-regulation of the pro-inflammatory proteins such as cytokines (IL-1β and TNF-α) and chemokines. It was also reported that the treatment with RGP decreased ROS levels [45]. 

### 5.4. White Grape Pomace Anti-Inflammatory Activity

Like in the case of antioxidant action, the anti-inflammatory activity of WGP is much less studied than RGP. In fact, almost all the studies focused on GP anti-inflammatory activity are done on RGP. We found only one article investigating WGP polyphenol extracts in vitro anti-inflammatory properties. According to this study, Ferri et al., (2017) used a bioluminescent cell-based assay to test WGP water and thanolic polyphenol extracts on human embryonic kidney HEK293 cells. It was observed that the water WGP extract reduced TNFa-induced inflammation by 62% [49].

These results support that GP possesses important antioxidant and anti-inflammatory activities. Although most studies are referring to RGP, there are insufficient data to conclude that it would be more effective than WGP, and further studies are needed to compare their activities.

## 6. Red and White Grape Pomace—In Vivo Antioxidant and Anti-Inflammatory Activities

Taking into consideration that several studies have demonstrated a beneficial impact on metabolic syndrome, which is a key factor in many health-related issues [50,51,52,53,54,55], for reducing cardiovascular disease risk factors such as TMAO (trimethylamine N-oxide) [56], hypertension, and hyperglycemia [57], it is necessary to find out if all of these are supported by in vivo studies. Thus, the literature can provide valuable information about the anti-inflammatory and antioxidant activity that GP possesses in various experimental studies [58,59,60].

The liver is the main antioxidant site for neutralizing most oxygen-free radicals. For this reason, the liver also plays a key role in maintaining the oxidant/antioxidant balance. This balance can be disturbed by diseases such as atherosclerosis, diabetes, and cancer, which induce oxidative stress. This state occurs when there is an increase in the production of free radicals, which can damage biomolecules such as lipids (lipid peroxidation), proteins (peptide chain fragmentation and electrical charge alteration), and DNA (purine and pyrimidine bases degradation, mutations, translocations or deletion) [61]. An additional measure in combating these pathological changes is brought by antioxidants, including GP, which is known to have a strong antioxidant effect. Thus, many studies have aimed to investigate the effects of GP on liver redox homeostasis. 

### 6.1. Red Grape Pomace Antioxidant Activity

One such study investigated the effect of RGP obtained from *Vitis vinifera* L. var. Moschato from Tyrnavos, Larissa, Greece on a batch of 36 Chios breed sheep [60]. Kerasioti et al., (2017) observed that this extract possesses an intense antioxidant activity demonstrated by increasing GST (glutathione transferase) activity and γ-GCS (γ-synthase glutamyl cysteine) expression in the liver [60]. Both GST and γ-GCS are enzymes involved in the synthesis of GSH (glutathione), which represents an important endogenous antioxidant. The antioxidant and anti-inflammatory activity of RGP obtained from *Vitis vinifera* from Uva’Só, Garibaldi, Rio Grande do Sul state, Brazil was also observed by Souza et al., (2019). Within their study, the effect of RGP was investigated on a model of liver damage induced by Pseudomonas aeruginosa in juvenile grass carps. The study highlighted the fact that the RGP possesses antioxidant activity as demonstrated by the reduction of TBARS levels, NOx, and ROS production [58]. Another study that contributed to the strengthening of the hypothesis that GP has a beneficial role in maintaining the oxidant/antioxidant balance is the study by Chedea et al., (2019). Their research was focused on the effects of RGP from *Vitis vinifera* from Valea Călugărească, România on 20 crossbred TOPIG hybrid pigs. They observed that administration of RGP had antioxidant activity as a result of the increased SOD (superoxide dismutase) activity and reduced TBARS levels [59]. SOD is an antioxidant enzyme that catalyzes the dismutation of the superoxide radical into oxygen and hydrogen peroxide. Even though hydrogen peroxide is still an oxygen-free radical, it is degraded by other antioxidant enzymes such as CAT (catalase) [62], whose activity is also increased by RGP [59,63,64]. Besides the antioxidant activity shown in the liver, studies reported that GP can also act on other organs. Thus, the administration of RGP increased SOD and CAT activity in the spleen and kidney [59], increased SOD; CAT, and GPx (glutathione peroxidase) activity; and reduced TBARS levels in the duodenum [64].

### 6.2. Red Grapepomace Anti-Inflammatory Activity

It is well-known that oxidative stress, via free radicals, induces damage to different cells and tissues, which leads to an inflammatory response from the organism but, besides this, alternative pathways have also been reported, which could prove the direct anti-inflammatory activity. One such study is that of Boussenna et al., (2016) who investigated the anti-inflammatory activity of GPs from *Vitis vinifera* from the Rhône valley, France on dextran sodium sulfate-induced inflammatory bowel disease in mice. They underlined that GP reduced polymorphonuclear (PMN) infiltration and attenuated the intensity and breadth of colonic modifications [65]. Another pro-inflammatory pathway where GP seemed to have an antagonistic effect is represented by the NF-κB activation. NF-κB represents an inflammatory factor that possesses a central role in different pro-inflammatory signaling pathways, such as transcriptional induction of cytokines and chemokines; promoting T cells differentiation [66]; activating macrophages which produce pro-inflammatory cytokines (IL-1, IL-6, IL-12, and TNF- α) [67,68]. Thus, Nishiumi et al., (2012) observed that GP from *Vitis vinifera* from Kobe City, Japan inhibited the activation of NF-κB, which led to a suppression of the expression of COX-2 (cyclooxygenases-2) and iNOS (inducible nitric oxide synthase) proteins. iNOS leads to an excess of NO, which could act as a free radical and COX-2 produces prostacyclins and prostaglandins, which are pro-inflammatory mediators. Thus, both suppressive actions demonstrate the anti-inflammatory activity of RGP [69]. The direct anti-inflammatory effect of RGP was observed on obese-induced C57BLK/6J mice [70]. This study suggests that GP anti-inflammatory effects could be enhanced by protective mechanisms other than the antioxidant activity demonstrated in other research works [70].

### 6.3. White Grape Pomace Antioxidant and Anti-Inflammatory Activities

So far, to our knowledge, the antioxidant and anti-inflammatory activity of WGP was not studied. However, two studies that compared the antioxidant and anti-inflammatory activities of RGP and WGP were identified. Nishiumi et al., (2012) studied RGP and WGP from *Vitis vinifera* grown in Kobe City, Japan. They evaluated the galactosamine and lipopolysaccharide-induced inflammation in 6-week-old Sprague-Dawley males. It was observed that RGP possessed a higher anti-inflammatory activity demonstrated by a stronger NF-κB inhibition as compared to the inhibitory action of WGP [69]. The second one, conducted by Turcu et al., (2020), studied the antioxidant activity of RGP from the Merlot variety and WGP from the Tămâioasă Românească variety. They observed that both 3% and 6% concentrations of both RGP and WGP reduced TBARS levels in tight meat, but only 3% WGP and 6% RGP reduced TBARS levels in breast meat. This could suggest the fact that WGP has higher antioxidant activity, requiring a lower concentration compared to RGP to induce the same effect [71]. The following table (Table 3) presents the antioxidant and anti-inflammatory activity of RGP alone and of RGP in comparison with WGP.

Unfortunately, most studies in the literature focused on the antioxidant and anti-inflammatory activity of GP do not specify whether it comes from red or white grapes. At the same time, in most of the experiments, red grapes were much more often used, so it is difficult to highlight whether there is a difference between the antioxidant and anti-inflammatory activity between RGP and WGP. For these reasons, there is a need for future studies addressing this topic to specify more accurately the origin of the GP. Moreover, it is well recommended that future studies should conduct a comparison between RGP and WGP, in order to highlight whether or not there are differences between their antioxidant and anti-inflammatory activity, and, if there are some, to be able to determine which of the two possesses a more potent activity.

## 7. Ischemic Heart Diseases—What We Know So Far and What Can Be Improved

When it comes to ischemic heart diseases, the literature refers to them as coronary artery diseases (CAD). Thereby, CAD is characterized as a pathological process caused by the accumulation of atherosclerotic plaque in the intimal wall of the arteries. This accumulation could lead to a complete or incomplete obstruction of the arteries, resulting in an imbalance between myocardial oxygen demand and supply. Other causes that could induce CAD are microvascular dysfunction and a spasm of the coronary arteries. This pathology is considered to be a chronic and progressive disease [2,72].

### 7.1. Risk Factors

The principal risk factors that contribute to the appearance and progression of atherosclerosis and coronary artery diseases are smoking, diet, weight gain, and physical activity. Among risk factors, tobacco is responsible for more than 8 million death per year. According to WHO, over 80% of tobacco users are from low-middle-income countries, and, taking into consideration that 75% of total deaths of cardiovascular events are registered also in low- and middle-income countries, there is a possible correlation between smoking and cardiovascular death causes. It was observed that smoking cessation leads to a reduction of 36% in CAD-induced mortality. For this matter, besides behavioral counseling, there is also pharmacological support to encourage smoking cessation [72,73]. Another risk factor is represented by an unhealthy diet. For the prevention of several diseases, a dietary plan that includes fruits, vegetables, polyunsaturated fats, fish, and fiber is recommended, along with avoiding a high quantity of refined carbohydrates, saturated, fat and red meat [72]. Nonetheless, physical inactivity represents a major risk factor for CAD and stroke. Both diet and physical activity modulate weight management, which represents one of the most life-long risks of all because a close correlation between body weight and lipid profile has been demonstrated in several studies. Increased body weight may also disturbs the lipid profile, which, could lead to atherosclerosis. According to WHO, over 39 million children under 5 years are obese, and over 1.9 billion adults are overweight, of which over 650 million are obese [74]. Therefore, it was demonstrated that patients who are overweight or obese are more likely to develop cardiovascular diseases than patients with a normal BMI (20–25 kg/m^2^) [72]. Considering these, a healthy lifestyle behavior would decrease the risk of cardiovascular events.

### 7.2. Diagnostics

When it comes to diagnostic methods, there is basic testing, which includes resting ECG, and echocardiography and biochemical tests such as a lipid profile and myocardial injury markers—troponins T and I. In addition to imagistic testing, if the echocardiography is inconclusive cardiac magnetic resonance may be taken into consideration [72]. In the last years, the medical scientific community tried to develop a way for less invasive and less expensive screening for this pathology. In this direction, a risk-estimation system was created and validated, the well-known SCORE system [72].

### 7.3. Management

The aims of pharmacological management are to reduce the symptoms associated with coronary artery diseases and to prevent major acute cardiovascular events like myocardial infarction. Starting from here, there are two types of therapy: for and for no life-threatening CAD, the second one is usually referred to it as long-term medication. For life-threatening CAD, the gold standard is percutaneous coronary intervention, within 2 h from the appearance of symptoms associated with antiplatelet and anticoagulant therapy. An alternative to percutaneous coronary intervention is fibrinolystic drugs such as alteplase or reteplase. The long-term medication includes numerous classes of drugs such as: anti-ischemic drugs, which include nitrates, beta-blockers, and calcium channel blockers; antiplatelet drugs like aspirin and clopidogrel; anticoagulant drugs, which include heparin and low molecular weight heparins, warfarin, dabigatran. All of these treatments come with side effects such as hypotension, headache (nitrates), fatigue, bradycardia, bronchospasm, heart block, peripheral vasoconstriction (beta-blockers), headache and ankle edema (calcium channel blockers), and increased risk of bleeding (antiplatelet and anticoagulant drugs) [72,75].

### 7.4. Potential New Therapy

Taking all of the above into consideration, even with the fact that the major risk factors are well-known and there is a continuous progression in diagnostic methods and pharmacological management, ischemic heart diseases remain one of the major causes of mortality and morbidity so far. Accordingly, there still exists the demand and necessity for alternative therapy to be found. That is why, when taking into consideration the oxidative stress and inflammation associated with all the mechanisms that induce ischemic heart diseases, it is a good premise that the polyphenols from GP could be used as adjuvant therapy. Even though the antioxidant and anti-inflammatory effects of the polyphenols from GP are well-known, there is still a lack of information when it comes to the pharmacological activities, especially their pharmacokinetics and pharmacodynamics. Looking in this direction, it is necessary to conduct studies for this purpose, firstly on animals, and, if the results are promising, to take the next step, and, eventually, to conduct trials on patients with ischemic heart diseases.

## 8. In Vitro and In Vivo Studies—Grape Pomace Antioxidant and Anti-Inflammatory Actions in Ischemic Heart Diseases

As was already mentioned, GP represents a reliable source of polyphenols that could be used as adjuvant therapies in the treatment of different pathologies characterized by oxidative stress or inflammatory pathophysiological processes. Moreover, GP proved that it could be used in various cardiac diseases due to its antioxidant and anti-inflammatory activities as observed in both in-vitro and in-vivo studies (Table 4). Grape pomace showed beneficial properties both when used as primary prophylaxis, in the control of risk factors, and when used as secondary prophylaxis, in the prevention of complications.

**Figure 3:**
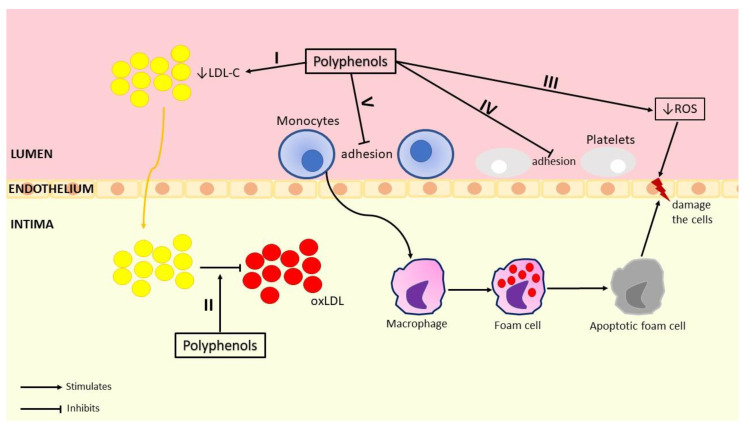
Anti-atherogenic effect of polyphenols from grape pomace (I—Polyphenols reduce LDL levels; II—Polyphenols reduce oxLDL formation; III—Polyphenols reduce ROS production; IV—Polyphenols inhibit platelets activation and adhesion by blocking its agonists; V—Polyphenols reduce monocytes adhesion by reducing the expression of VCAM-1).

Used as primary prophylaxis, it was observed that the polyphenols from GP presented a protective effect by their anti-atherogenic actions. It is well known that a key factor in the progression of atherosclerosis is the oxidation of LDL cholesterol and the fact that atherosclerosis triggers an inflammatory response in the arterial wall (Figure 3). Despite the existence of lipid-lowering drug classes, statins, and fibrates, which decrease LDL cholesterol, total cholesterol, and triglycerides levels and increase HDL cholesterol levels, there is still a large majority of patients who develop ischemic heart diseases [76,77]. So theoretically, GP due to its antioxidant activity could help in the management of atherosclerosis development and progression by its capacity to prevent LDL cholesterol oxidation and by its anti-inflammatory activity. With this perspective, Rivera et al., (2019) studied the effect of RGP on ischemic heart disease in rats with induced atherosclerosis through an atherogenic diet. They observed that RGP presented anti-inflammatory activity by decreasing TNF-α and IL-10 levels. Moreover, they observed that the RGP added to the diet also increased the concentration of HDL cholesterol, which has an anti-atherogenic effect. Even more, in comparison with the control group, the RGP decreased the size and number of atherosclerotic lesions [78]. Other in vitro and in vivo studies also showed that GP possesses antioxidant activity useful in the management of the prooxidant status, considered a key factor in atherosclerosis development. Thus, GP can increase SOD, CAT, GPx, and/or GRx levels [79,80,81,82] and reduce ROS GSH, TBARS, and/or superoxide levels [83,84,85] being useful in primary prophylaxis.

Another key factor in the atherosclerosis process is represented by the platelet aggregation induced by endothelium dysfunctions. Muñoz-Bernal et al., (2021) studied the effect of GP from nine types of *Vitis vinifera* on platelet aggregation induced by adenosine diphosphate (ADP), which activates aggregation acting on several receptors like P2Y1, P2Y12, P2 × 1, an ATPC receptor, and by thrombin receptor activating peptide 6 (TRAP-6), which bind to the thrombin receptor. They observed that only the GP from Petit Verdot possesses anti-platelet aggregation, with a 67.1% inhibition of ADP-induced platelets aggregation and a 53.2% inhibition of TRAP-6 [86]. The same inhibitory effect on ADP-induced platelets aggregation (Figure 3) was observed by Bijak et al., (2019), who highlighted not just the anti-platelet effect, but also an anticoagulant effect of GP from *Vitis vinifera* from Hamburg, Germany [87].

Further, if not all the non-pharmacological and pharmacological interventions are successful in atherosclerosis optimum management, in time, the patients are predisposed to develop chronic ischemic heart disease and sometimes acute events. Therefore, patients will need secondary prophylaxis, aiming to prevent the occurrence of complications. Within this context, due to the antioxidant activity presented above, useful in preventing the continuous development of pre-existing atherosclerosis, GP represents a valuable source of polyphenols for CAD secondary prophylaxis. 

The prevention of athero-thrombotic episodes that may occur in patients with CAD is also crucial. Therefore, Carrieri et al., (2013) studied the effects of GP from 12 grape varieties on LPS-induced tissue factor (TF) activity [88]. TF is a key factor in the pathogenesis of many thrombotic diseases because of its role in the initiation of coagulation via the extrinsic pathway. TF synthesis is modulated by monocytes and macrophages and activated by different inflammatory factors [89,90]. They observed that GP possessed an inhibitor activity on LPS-induced TF activity and, in general, RGP had the highest activity on both blood and on mononuclear cells. Furthermore, they identified a positive correlation between the concentration of quercetin, cyanidin, and TF inhibition. Moreover, they also identified a negative correlation between the concentration of malvidin, myricetin, petunidin, and TF inhibition [88]. Xuan et al., (2012) observed that in mice with surgical-induced myocardial infarction, resveratrol improves the survival rate from 55% to 80% through several actions. The resveratrol-treated group had a smaller left ventricle infarct size. Moreover, echocardiography highlighted a decrease in cardiac remodeling proven by a smaller left ventricular end-systolic and end-diastolic diameter and a larger left ventricular fractional shortening [91]. Das et al., (2014) also observed the cardioprotective effects of resveratrol in their study performed on mice under ischemic/reperfusion exposure. Besides the antioxidant effects demonstrated by decreased ROS and GSH levels, a reduction in the infarct size and an improvement in left ventricular developed pressure were also observed. Even more, an improvement in the aortic flow, which is an effect of left ventricular improvement, was observed [92]. The same left ventricular improvement was described by Rivera et al., (2019), who observed a restoration in the left ventricle’s ejection fraction in the myocardial infarction studied in atherogenic diet-induced mice fed with RGP [78]. The cardioprotective effects as well as antioxidant and anti-inflammatory actions of polyphenols and GP in different experimental settings are summarized in Table 4.

All these antioxidant, anti-inflammatory, and cardioprotective effects are a solid argument for further research concerning GP’s potential therapeutic effect in the treatment of patients with ischemic heart disease. In addition to this, it is necessary to identify if it has significant positive effects as primary prophylaxis because it is much more important to prevent the occurrence of the disease by controlling risk factors than preventing the occurrence of complications by controlling the underlying pathology. Thus, it is necessary to compare these effects between RGP and WGP to identify the best substrate that can be used as a future therapy.

**Table 4:** The cardioprotective effects, antioxidant, and anti-inflammatory actions of polyphenols and grape pomace on different cardiac experimental injuries.

*In Vitro* Studies			
**Materials**	Models	Antioxidant and Anti-Inflammatory Effects	References
Resveratrol—100 μmol/L	Neonatal cardiac cells under ischemia/reperfusion exposure	- increased Bcl-2 expression - increased SOD levels - increased cell viability - increased the activity of Ca2+ -ATPase and Na+-K+ -ATPase - reduced the activity of caspase-3 and LDH - reduced apoptotic rate - reduced Bax expression - reduced MDA levels	[79]
Resveratrol—1 mL/2.5 mg/kg food	H_2_O_2_ exposed cardiomyocytes sampled from Sprague Dawley rats	- averted the activity reduction of CAT and SOD	[80]
Resveratrol—3 μM	Human cardiomyocytes’ azidothymidine-induced cardiotoxicity	- decreased the activity of caspase-3, caspase-7 - decreased mitochondrial ROS generation - decreased cardiomyocytes apoptosis	[83]
Resveratrol—3 μM	Neonatal human cardiomyocytes in a medium with endotoxin lipopolysaccharide	- decreased the mitochondrial production of ROS	[84]
Grape pomace from Vitis vinifera L. cv. Barbera, Carignan, Cabernet Sauvignon, Grenache, Merlot, Petit Verdot, Syrah, Tempranillo and Zinfandel from Baja California, Mexico/methanol extract	Human platelets from 6 healthy persons	- GP from Petit Verdot possessed significant anti-platelets aggregation induced by ADP and TRAP-6	[86]
Grape pomace from Vitis vinifera from Hamburg, Germany	Blood samples	- inhibited the ADP-induced platelets aggregation - anticoagulant effect–prolonged activated partial - thromboplastin time (APPT) and prothrombin time (PT)	[87]
12 GP from white (Italia, Baresana, Beogradska, Autumn Seedless), red (Crimson Seedless, Red Globe, Apulia Rose, Supernova) and black (Autumn Royal, Michele Palieri, Summer Royal) from Turi, Italy	Blood samples from healthy donors Mononuclear cells isolated from the blood samples	- on blood: inhibited the LPS-induced TF activity, where RGP had the highest inhibition rate, followed by WGP and BGP - on mononuclear cells: inhibited the PLS-induced TF activity expression, where Supernova (red) had the highest inhibition rate	[88]
Resveratrol—25 μM	Neonatal rat cardiac cells in a medium with fractalkine	- conserved cardiac cell viability - augmented cardiac cells autophagy - decreased levels of MMP-9, ANP, ICAM-1, TGF-β, FKN, procollagens I and III	[91]
***In vivo* studies**			
Red grape pomace—10% concentration	60 rats with atherogenic diet-induced ischemic heart disease	- decreased levels of IL-10 and TNF-α - decreased the number and dimension of atherosclerotic lesions - increased levels of HDL cholesterol - restored the ejection fraction in the left ventricle	[78]
Grape skin and seed extract from *Vitis vinifera* cultivated in northern Tunisia—4 g/kg	24 Wistar male rats with cardiac injury and oxidative stress induced by arsenic	- increased SOD, GPx and CAT activities - reduced the levels of free iron, ionizable calcium and H_2_O_2_	[81]
Grape seed proanthocyanidins—100 mg/kg, twice a day	Ischemic-induced left ventricle by a 0.09% NaCl–4oC solution from 32 Rattus Norvegicus rats	- reduced ischemia-related MDA - increased SOD, GPx and CAT levels	[82]
Resveratrol—10 mg/kg IP injection	C57BL/6 mice with endotoxin-induced cardiomyopathy	- reduced the increase of CK and LDH levels	[84]
Resveratrol—10 mg/kg/day	L-NAME-induced malignant hypertension mice	- decreased superoxide anion radical, TBARs, MPO, thiol groups - decreased the damage score of cardiomyocytes - decreased expression of TGF-β - increased NO2 - averted reduction of antioxidant enzymes: SOD, GRx, GPx, CAT	[85]
Resveratrol—20 mg/kg IP injection for 42 days * IP—intraperitoneal injection	C57BL/6 mice with myocardial infarction surgical-induced	- increased the function of the left ventricle - increased survival rate - reduced the infarction size of the left ventricle	[91]
Resveratrol—2.5 mg/kg for 15 days	Rats under ischemic/reperfusion exposure	- decreased infarction size - reduced ROS and GSH levels - increased aortic flow - increased the development of left ventricular pressure post-ischemia	[92]
Methanolic extract from *Vitis vinifera* seed—125/250 mg/kg	Isoproterenol-induced infarction and streptozotocin-induced diabetes in Wistar mice	- decreased LPO levels - decreased expression of RAGE protein - decreased TNF-α, IL-1β, IL-6, NF- κβ and Iκκβ levels - increased CAT, GPx and SOD levels - increased the activity of Ca2+ -ATPase and Na+-K+ -ATPase	[93]
Resveratrol—30/100 mg/kg for 7 days	Heart sampled from ApoE-KO rats	- decreased levels of ROS and superoxide - decreased expression of NOX2 and NOX 4, with no effect on NOX1 - increased expression of SOD (SOD1, SOD2 and SOD3), catalase and GSH-Px	[94]

**Abbreviations:** ROS—Reactive oxygen species; ICAM-1—Intercellular adhesion molecule 1; MMP-9—Matrix metallopeptidase 9; FKN—Fractalkine; SOD—Superoxide dismutase; LDH—Lactate dehydrogenase; MDA—Malondialdehyde; CAT—catalase; ANP—Atrial natriuretic peptide; TGF-β—Transforming growth factor beta; GPx/GSH-Px—Glutathione peroxidase; LPO—Lipid peroxidation; IL-10—Interleukin-10; TNF-α—Tumor necrosis factor alpha; HDL—High-density lipoprotein; NF-kb—Nuclear factor kappa-light-chain-enhancer of activated B cells; Ikkb—Inhibitor of nuclear factor kappa-B kinase subunit beta; IL-1b—interleukin-1b; IL-6, interleukin-6; TBARs—Thiobarbituric acid reactive substances; MPO—Myeloperoxidase; GSH—Glutathione; GRx—Glutathione reductase; NOX – nitrate oxide.

## 9. Conclusions

The potential of polyphenols from GP is well known because of their antioxidant and anti-inflammatory effects. However, the existing data concerning total phenolic content and antioxidant activity according to GP source (red or white grapes) is still limited and there is a clear need to underline the differences between RGP and WGP. Therefore, as future direction, it is necessary to evaluate the geographical, climacteric, and preparation factors of RGP and WGP, using common and standardized bioactive compound quantification methods to establish the differences. These differences are needed to give a proper valorization of them in CAD prophylaxis through antioxidant and anti-inflammatory actions. Furthermore, there are still no sufficient studies to compare RGP and WGP activities in humans, in order to identify the better one to be used in CAD.

In conclusion, knowing that GP presents cardioprotective effects in ischemic heart diseases by modulating and decreasing both atherosclerosis development and atherosclerotic lesions and preserving cardiac function, the development of adjuvant therapy based on GP for patients with cardiovascular diseases would be helpful. 

## Figures and Tables

**Table 2:** In vitro antioxidant and anti-inflammatory activity of grape pomace polyphenols extracts.

Materials	Polyphenols Extracts	Models	Antioxidant and Anti-Inflammatory Activity	References
Grape pomace from different red *Vitis vinifera* species				
GP from *Vitis vinifera* L. Cagnulari cv. from Santa Maria La Palma, Alghero, Italy	Water/ethanol (60:40, *v*/*v*) extract containing: - anthocyanins (malvidin, peonidin-3-O-glucoside, malvidin-3-(6-acetyl)-glucoside, M-3-G)	H_2_O_2_-induced oxidative damage in human umbilical vein endothelial cells	- increased cells viability - reduced ROS levels	[39]
GP from *Vitis vinifera* L. Batiki Tyrnavou cv. from Greece	Ethanol extract containing: - flavan-3-ols (catechin, epicatechin, epicatechin-3-gallate) - anthocyanidins (malvidin, cyanidin, petunidin, delphinidin) - anthocyanins (peonidin-3-O-glucoside, myrtillin, oenin, kuromanin) - phenolic acids (caftaric acid, gallic acid) - flavanols (quercetin, kaempferol)	Tert-butyl hydroperoxide-induced oxidative damage in muscle cells (C2C12)	- reduced TBARS, ROS and protein carbonyls levels - increased GSH levels	[40]
		Tert-butyl hydroperoxide-induced oxidative damage in endothelial cells (EA.hy926)	- reduced TBARS and protein carbonyls levels - increased GSH levels	
GP from *Vitis vinifera* seeds	-	UV radiation-induced oxidative stress in human keratinocytes cells (HaCaT cells)	- decreased ROS levels - decreased apoptosis proteins levels - decreased Bax-α pro-apoptotic protein levels - decreased NF-kB p65 protein levels	[41]
GP from *Vitis vinifera* from Valea Calugareasca	Acetone extract containing: - flavonoids (catechins, procyanidins, epicatechins) * higher concentration for procyanidin dimer and epicatechin	Intestinal inflammation model: LPS-inflammation induced in Caco-2 intestinal cells Symbiotic combination with *Lactobacillus sp*. as probiotic	- down-regulation of chemokines and cytokines proteins and genes expression - up-regulation of TIMP1 and TIMP2 genes expression - down-regulation of JNK1, ERK1/2, Akt/P70S6K/mTOR, MAPK, NF-κB and Nrf2 expression	[42]
GP from *Vitis vinifera* variety Montepulciano from Chieti, Italy	Water extract containing: - gallic acid, caftaric acid, caffeic aicd, syringic acid, coumaric acid, ferulic acid, catechin, epicatechin, chlorogenic acid	H_2_O_2_-induced oxidative damage in HypoE22 rat hypothalamus cells	- averted the down-regulation of BDNF gene expression - averted up-regulation of COX-2 gene expression and decreased PGE2 levels	[43]
GP from *Vitis vinifera* L. varieties from Emilia Romagna region, Italy	Natural deep eutectic solvents (NaDESs) extract containing: - anthocyanins (malvidin)	Menadione-induced oxidative damage in keratinocyte cells from human skin (HaCaT cells)	- improved cells viability - reduced IL-8 levels	[44]
GP from *Vitis vinifera* L., cv Negramaro from Azienda Agricola Cantele, Guagnano, Lecce, Italy	Methanol/ethanol (80:20, *v*/*v*) extract containing: - caffeic acid, caftraic acid, cutaric acid, gallic acid, catechin, epicatechin, kampferol, oenin, quercetin, rutin, t-resveratrol	LPS and TNF-α-induced inflammation in human colorectal adenocarcinoma-derived intestinal epithelial cells (Caco-2 cells) and human microvascular endothelial cells (HMEC-1 cells)	- decreased IL-6 and MCP-1 levels - down-regulation of MMP-9 and MMP-2 expression - down-regulation of the mRNA levels of the cytokines (IL-1β and TNF-α), the chemokines (CXCL-10 and M-CSF), COX-2 VCAM-1, ICAM-1 - down-regulation of the NF-κB signaling pathways - reduced ROS levels	[45]
GP from *Vitis vinifera* cv Pinot noir from Cautín valley, La Araucanía Region, Chile	Ethanol extract containing: - hydroxybenzoic acids (gallic acid, protocatechuic acid) - flavanol (catechin) - hydroxycinnamic acid (ferulic acid) - flavanols (quercetin, quercetin-3-rutinoside, quercetin-3-galactoside, quercetin-3-glucoside, kaempferol-3-glucoside) - anthocyanins (malvidin-3-glucoside, peonidin-3-glucoside, delphinin-3-glucoside, petunidin-3-glucoside, cyanidin-3-glucoside)	Polycyclic aromatic hydrocarbons-induced cytotoxicity in endothelial cells	- increased cells viability - down-regulation of Nrf2 expression	[46]
Grape pomace from different white *Vitis vinifera* species				
GP from *Vitis vinifera* cv Chardonnay from Lowden, WA, USA	-	H_2_O_2_-induced oxidative damage in human colonic epithelial cells (Caco-2 cells)	- decreased ROS levels	[47]
Red grape pomace versus White grape pomace				
GP from *Vitis vinifera* varieties from Quinta da Cavadinha, Pinhão, Portugal Red: Tinto Cão, Tinta Barroca White: Malvasia Fina, Moscatel Branco	Methanol/distilled water (70:30, *v*/*v*) extract containing: - flavanols (isorhamnetin-3- O-(6-O-feruloyl)-glucoside, quercetin-3-O- glucuronide, quercetin-3-O-rutinoside, kaempferol-3-O-rutinoside, kaempferol-3-O-gluco- side) - cinnamic acid (caftaric acid) - anthocyanins (malvidin-3-O-glucoside, malvidin-3-O-(6-O-caffeoyl)-glucoside, malvidin-3-O-rutinoside) - stilbene (Σ-viniferin)	H_2_O_2_-induced oxidative damage in human keratinocytes (HaCaT cells)	- increased GSH levels, where WGP from Malvasia Fina had the highest capacity to increase it - decreased ROS levels, where RGP from Tinto Cão had the highest capacity to decrease it - decreased LPO levels, where WGP from Malvasia Fina had the highest capacity to decrease it	[48]

**Abbreviations:** TIMP1/2—matrix metalloproteinase inhibitors 1/2; MAPK—Mitogen-activated protein kinase; JNK1—c-Jun N-terminal kinase; ERK1/2—Extracellular signal-regulated kinase 1/2; Akt—protein kinase B; P70S6K—ribosomal protein S6 kinase; mTOR—mammalian target of rapamycin; Nrf2—nuclear factor erythroid 2–related factor 2; ROS– Reactive oxygen species; TBARS- Thiobarbituric acid reactive substances; GSH –Glutathione; BDNF—brain-derived neurotrophic factor; PGE2—Prostaglandin E2; LPO—Lipid peroxidation; NF-kB –nuclear factor kappa-light-chain-enhancer of activated B cells; LPS—lipopolysaccharide; TNF-α—tumor necrosis factor; IL-6—Interleukin-6; MCP-1—monocyte chemoattractant protein-1; MMP—matrix metalloproteinases; IL-1β—Interleukin-1-beta; CXCL-10—C-X-C motif chemokine ligand 10; M-CSF—macrophage colony-stimulating factor; COX-2– cyclooxygenase-2; VCAM-1– Vascular Cell Adhesion Molecule 1; ICAM-1– Intercellular Adhesion Molecule 1; H_2_O_2_—hydrogen peroxide.

**Table 3:** In vivo antioxidant and anti-inflammatory activity of grape pomace polyphenols extracts.

Materials	Polyphenols Extracts	Models	Antioxidant and Anti-Inflammatory Activity	References
Grape pomace from different red *Vitis vinifera* cultivars				
*Vitis vinifera* sp. Cabernet Franc from Blackstone, VA, USA	Ethanol extract	Streptozotocin-induced type 2 diabetes in 6-week-old C57BL/6J male mice	- suppressed the rising of postprandial blood glucose	[36]
*Vitis vinifera* from Uva’Só, Garibaldi, Rio Grande do Sul state, Brazil	-	Pseudomonas aeruginosa-induced hepatic lesion in juvenile grass carps	- reduced NOx and ROS production - reduced TBARS levels - reduced the increase of SOD and CAT activity through their antioxidant activity - no significant increase in GPx and GST activities	[58]
*Vitis vinifera* from Valea Calugareasca, Romania	Methanol/Acetone extracts	Organs (liver, spleen, kidney) sampled from 20 crossbred TOPIG hybrid (Landrace & Large White with Duroc & Pietrain) pigs	- increased SOD activity and total antioxidant status in all of these organs - significantly increased CAT activity in kidney and spleen - no significant difference in GPx activity - significantly reduced TBARS levels in liver and kidney	[59]
*Vitis vinifera* L. var. Moschato from Tyrnavos (Larissa, Greece)	Water extract	36 Chios breed male sheep	- increased GST activity in the spleen and liver - increased γ-GCS expression in liver - no significant difference in SOD activity in both liver and spleen	[60]
*Vitis labruscana* L. from Korea	Methanol and ethanol extract	Diet-induced hypercholesterolemia in 48 New Zealand white male rabbits	- increased CAT and GPx activity - significantly reduced TBARS levels	[63]
*Vitis vinifera* from Valea Călugaărească, România	Water extract containing: - procyanidin trimer, procyanidin dimer, gallic acid, gallic acid-glucoside, malvidin-3-O-(6″-coumaroyl-glucoside)	Duodenum and Colon sampled from 20 crossbred TOPIG hybrid (Landrace & Large White with Duroc & Pietrain) pigs	- significantly reduced TBARS levels - increased total antioxidant status - increased SOD activity only in the duodenum - increased GPx and CAT activity	[64]
*Vitis vinifera* from the Rhône valley, France	Ethanol extract containing: - anthocyanins (malvidin-3-glucoside)	Dextran sodium sulfate-induced inflammatory bowel disease in 5-week-old Wistar male mice	- increased SOD activity - decreased PMN infiltration	[65]
*Vitis vinifera* from Kobe City, Japan	1% acetic acid in methanol extract	Galactosamine and lipopolysaccharide-induced inflammation in 6-week-old Sprague-Dawley male	- suppress the expressions of COX-2 and iNOS proteins by inhibiting NF-κB activation	[69]
*Vitis vinifera* from Virginia vineyard (Blackstone, VA, USA)	Ethanol 1:10 ratio (*m/v*) extract containing: - catechin, epicatechin, quercetin, trans-resveratrol, caffeic acid, coutaric acid, ferulic acid, gallic acid, p-coumaric acid, p-hydroxybenzoic acid, protocatechuic acid, syringic acid	Obese 6 -week-old C57BLK/6J male mice	- no significant antioxidant activity - anti-inflammatory activity: reduced CRP levels	[70]
Red grape pomace versus White grape pomace				
*Vitis vinifera* from Kobe City, Japan	1% acetic acid in methanol extract	Galactosamine and lipopolysaccharide-induced inflammation in 6-week-old Sprague-Dawley male	- red grape pomace possessed a higher inhibiting action of NF-κB activation than white grape pomace	[69]
*Vitis vinifera* from Pietroasa, Buzău county, România PUFA enriched * WGP from Tămâioasă Românească variety * RGP from Merlot variety	3% and 6% dried GP diet	Breast and thigh meat sampled from broilers fed with 3% and 6% grape pomace	- both 3% and 6% diets significantly reduced TBARS levels in tight meat; - just the 3% white GP and the 6% red GP diets significantly reduced TBARS levels in breast meat	[71]

**Abbreviations:** CRP—C-reactive protein; NOx—Nitrate oxide; ROS—Reactive oxygen species; TBARS—Thiobarbituric reactive substances; SOD—Superoxide dismutase; CAT—Catalase; GPx—Glutathione Peroxidase; GST—Glutathione transferase; PMN—Polymorphonuclear; γ -GCS—γ-synthase glutamyl cysteine; COX-2—cyclooxygenases-2; iNOS—Inducible nitric oxide synthase; NF-kB—nuclear factor - kappa-light-chain-enhancer of activated B cells; PUFA—polyunsaturated fatty acids.

## Data Availability

Data are contained within the article.
